# The FBPase Encoding Gene *glpX* Is Required for Gluconeogenesis, Bacterial Proliferation and Division *In Vivo* of *Mycobacterium marinum*

**DOI:** 10.1371/journal.pone.0156663

**Published:** 2016-05-27

**Authors:** Jingfeng Tong, Lu Meng, Xinwei Wang, Lixia Liu, Liangdong Lyu, Chuan Wang, Yang Li, Qian Gao, Chen Yang, Chen Niu

**Affiliations:** 1 MOE & MOH Key Laboratory of Medical Molecular Virology, School of Basic Medical Sciences, Fudan University, Shanghai, China; 2 Institutes of Biomedical Sciences and Institute of Medical Microbiology, Fudan University, Shanghai, China; 3 Institut Pasteur of Shanghai, Chinese Academy of Sciences, Shanghai, China; 4 Key Laboratory of Synthetic Biology, Institute of Plant Physiology and Ecology, Shanghai Institutes for Biological Sciences, Chinese Academy of Sciences, Shanghai, China; IPBS, FRANCE

## Abstract

Lipids have been identified as important carbon sources for *Mycobacterium tuberculosis* (*Mtb*) to utilize *in vivo*. Thus gluconeogenesis bears a key role for *Mtb* to survive and replicate in host. A rate-limiting enzyme of gluconeogenesis, fructose 1, 6-bisphosphatase (FBPase) is encoded by the gene *glpX*. The functions of *glpX* were studied in *M*. *marinum*, a closely related species to *Mtb*. The *glpX* deletion strain (**Δ***glpX*) displayed altered gluconeogenesis, attenuated virulence, and altered bacterial proliferation. Metabolic profiles indicate an accumulation of the FBPase substrate, fructose 1, 6-bisphosphate (FBP) and altered gluconeogenic flux when Δ*glpX* is cultivated in a gluconeogenic carbon substrate, acetate. In both macrophages and zebrafish, the proliferation of Δ*glpX* was halted, resulting in dramatically attenuated virulence. Intracellular Δ*glpX* exhibited an elongated morphology, which was also observed when Δ*glpX* was grown in a gluconeogenic carbon source. This elongated morphology is also supported by the observation of unseparated multi-nucleoid cell, indicating that a complete mycobacterial division *in vivo* is correlated with intact gluconeogenesis. Together, our results indicate that *glpX* has essential functions in gluconeogenesis, and plays an indispensable role in bacterial proliferation *in vivo* and virulence of *M*. *marinum*.

## Introduction

*Mycobacterium tuberculosis* (*Mtb*), one of the most successful human pathogens, has evolved strategies to evade host immunity and cause tuberculosis (TB) [1,2]. During TB pathogenesis, central carbon metabolism (CCM) plays a critical role, and many CCM genes are considered essential [3]. Unlike *in vitro* cultivation, growing evidence indicates that *Mtb* tends to utilize lipids *in vivo*, including fatty acids, cholesterol, cholesteryl ester, and triacylglycerides [3,4]. Thus, both the glyoxylate cycle and gluconeogenesis have important roles in converting metabolites from lipid catabolism to key precursors in various bacterial physiological processes, such as the synthesis of peptidoglycan (PG) and amino acids [5,6]. Therefore, intensive studies have been performed on several genes in these metabolic pathways. For example, *icl1* and *icl2* from the *Mtb* genome encode isocitrate lysases in the glyoxylate cycle and are jointly required for *Mtb* survival in macrophages and mice through infection [7,8]. Moreover, *pckA* encoding phosphoenolpyruvate carboxykinase that catalyzes the conversion of oxaloacetate to phosphoenolpyruvate in gluconeogenesis plays a pivotal role for survival and proliferation of *Mtb* during mouse infection [9]. In addition, *fba* that modulates the switch between glycolysis and gluconeogenesis plays a considerable role in both acute and chronic mouse infections [10,11]. Therefore, gluconeogenesis is critical to TB pathogenesis and several gluconeogenic enzymes have been proposed as potential targets for chemotherapeutic interventions [10,11].

Fructose-1, 6-bisphosphatase (FBPase), encoded by *glpX*, is a key gluconeogenic enzyme catalyzing an irreversible reaction that converts fructose 1, 6-bisphosphate (FBP) to fructose 6-phosphate (F6P) [12,13]. F6P is a critical precursor for synthesis of cell envelope components, such as glycans and mannolipids [14,15,16]. Moreover, F6P is fed into the pentose phosphate pathway (PPP) for production of the precursors used in synthesis of nucleotides and aromatic amino acids. Thus, the roles of the FBPase encoding genes in CCM and virulence of *Mtb* has drawn much attention. An earlier report demonstrated that the overexpression of *Mtb glpX* rescued the growth defects of an *Escherichia coli* FBPase mutant [17]. In a recent report, *glpX* was demonstrated essential for both proliferation and virulence of *Mtb* [18]. In addition, gene *gpm2* (Rv3214) was identified as a novel FBPase [19], and the gluconeogenesis and virulence were seriously affected by the disruption of both *gpm2* and *glpX*. The importance of *glpX* in *Mtb* has been addressed through these studies. However, the mechanisms by which FBPase-encoding genes contribute to mycobacterial proliferation and virulence remains to be further investigated [19]. To address this issue, we performed a series of experiments using *M*. *marinum* which is a closely genetic relative to *Mtb*. The genomes between *Mtb* and *M*. *marinum* were compared earlier and display a high degree of homology [20]. Besides, as one of the natural hosts of *M*. *marinum*, zebrafish has been developed as an elegant model to investigate mycobacterial pathogenesis, especially in the angle of early TB pathogenesis [21,22,23].

In our study, a *glpX* deletion mutant (Δ*glpX*) of *M*. *marinum* was constructed, and the functions of *glpX* in carbon metabolism, bacterial proliferation and virulence were investigated through LC-MS analysis, and macrophage and zebrafish infections. The Δ*glpX* mutant displayed a disrupted gluconeogenesis when grown on gluconeogenic carbon source. Furthermore, *glpX* is required for virulence of *M*. *marinum* both in macrophages and zebrafish. The proliferation of Δ*glpX* was halted inside macrophage cells, and an elongated morphology appeared. We further sought the causes of this abnormal morphology and identified that *glpX* is required for intact division of *M*. *marinum* under gluconeogenic conditions and inside macrophage cells.

## Materials and Methods

### Strains and Culture Conditions

*M*. *marinum* M strain (ATCC BAA-535) was used as the wild type (WT) strain in this study. *M*. *marinum* was routinely cultivated in Middlebrook 7H9 broth or on 7H10 agar enriched with 10% OADC (oleic acid-albumin-dextrose-catalase) and 0.4% volume/volume (v/v) glycerol. When necessary, 40 μg/ml of kanamycin or 20 μg/ml of gentamycin or 50 μg/ml of hygromycin was included. For growth measurement, strains were cultured in 7H9 broth with or without 0.2% weight/volume (w/v) glucose, 0.2% v/v glycerol or 0.2% w/v acetate separately. In addition, 0.02% v/v tyloxapol was added into 7H9 broth to reduce bacterial clumping. Minimal medium is used for cultivation of *M*. *marinum* strains for LC-MS analysis, and the composition is as following: 1.5 g/L KH2PO4, 1.0 g/L NH4Cl, 0.2 g/L MgSO_4_·7H_2_O, 0.02 g/L CaCl_2_·2H_2_O, 0.85 g/L NaCl, 8.99 g/L Na_2_HPO_4_·12H_2_O, and 0.014 g/L (NH4)_2_ Fe (SO_4_)_2_.6H_2_O. The carbon sources supplemented into minimal medium are either 1 g/L glucose or 1 g/L acetate.

### The *glpX* Mutant Generation and Complementation

The *glpX* open reading frame (ORF) plus 1kb flanking region was PCR amplified from WT *M*. *marinum* genomic DNA using primers MM*glpX*KO_F and MM*glpX*KO_R, and the amplified fragment was ligated to T-vector. The 527–939 nt fragment of *glpX* was digested out with *SmaI*, and the hygromycin cassette from pHINT1 vector was inserted to obtain *glpX*::*hyg*, which was then digested with *SpeI* and cloned into *SpeI* site of the vector pPR27-*wasabi* (S1B Fig) to obtain pPR27*glpX*KO. The vector pPR27*glpX*KO was transformed into WT, and positive clones were first selected using hygromycin followed 10% w/v sucrose. Then, potential deletion mutants were selected by fluorescent microscopy for the loss of counter-selection marker *wasabi*. For complementation of Δ*glpX*, the MM*glpX* fragment containing the *glpX* gene together with 475 bp of upstream sequence was obtained by PCR amplification of *M*. *marinum* genomic DNA using primers MM*glpX*CO_F and MM*glpX*CO_R (S1 Table), and the fragment was digested with *EcoRI* and *SfiI*, ligated into *EcoRI* and *SfiI*-digested pMV306 vector, which is a single-copy attB integrating vector. The pMV306/C (*glpX*) vector was integrated into Δ*glpX* to get the complemented strain Δ*glpX*/C (*glpX*).

### LC-MS Analysis

*M*. *marinum* strains were precultured in 7H9 broth with 10% OADC to exponential phase. The cultures were started with an optical density at 600 nm (OD_600_) of 0.01 and performed in minimal medium with 1 g/L [U-^13^C] glucose or 1 g/L [U-^13^C] acetate. When OD_600_ reached 1.0, bacteria were collected by centrifugation and cell pellets were resuspended in 2 ml of 80:20 (v/v) methanol/water at -20°C separately and lysed by mechanical vortexing. After a centrifugation at 4°C for 3 min at 16200 ×*g*, the supernatants were collected. Metabolite and isotopomer analyses were performed with a UPLC system (Waters) coupled by negative mode electrospray ionization to an orbitrap mass spectrometer (Thermo Scientific). Injection volume was 20 μl. Solvent A was 50 mM ammonium acetate adjusted to pH 9.0 with ammonium hydroxide and solvent B was acetonitrile. Metabolites were separated with a Luna NH2 column (10-cm length, 2-mm diameter, 3-μm particle size; Phenomenex). The column was maintained at 15°C with a solvent flow rate of 0.2 ml min^-1^, and the gradient of B was as follows: 0 min, 85%; 3 min, 30%; 12 min, 2%; 15 min, 2%; 16 min, 85%; 23 min, 85%. The mass spectrometer scanned from *m*/*z* 70–1,000 at 1 Hz at 100,000 resolution. Data were analyzed using the Xcalibur software.

### Quantitative Real-Time PCR (qRT-PCR)

The transcriptional expression of selected genes was measured by qRT-PCR using primers listed in S1 Table. The gene *sigA* was applied as an internal control. The cDNA used in these experiments was prepared from RNA isolations obtained from three independent biological replicates. qRT-PCRs were carried out in triplicate using iQ SYBR green supermixture kit (Bio-Rad) and a CFX96^™^ Real-Time PCR System (Bio-Rad).

### Zebrafish Infection

Adult zebrafish were infected with M. marinum strains as described previously [24]. For larval infection, experiments were performed as described in a recent paper [25], and bacterial burdens in larvae were determined by fluorescence pixel counts (FPC) through the ImageJ software. Animal work was approved by IACUC of Fudan University (20120105–001). To minimize animal suffering and distress, adult or larval zebrafish were injected under anesthesia. The animal survival study was performed using adult fish, the specific criteria regarding humane endpoints is set as adult fish show irregularities in food consumption, hemorrhaging, and buoyancy loss, removal of animal whose illness or condition makes them nonproductive to the study. Fish are euthanized with an excess dosage of tricaine (MS-222) on ice, with a final concentration 0.2% w/v in fish water. Larval fish showed no signs of morbidity during the experimental period. We performed daily observations for fish health and mortality. The number of unexpected deaths during the experiment was zero. The dead or euthanized fish were disposed following the requirement of IACUC of Fudan University.

### Macrophage Infections and Length Measurement of Intracellular *M*. *marinum*

Raw264.7 macrophages were infected with *M*. *marinum* strains at multiplicity of infection (MOI) of 1, and intracellular bacterial load was counted as described in a recent paper [25]. For length measurement of intracellular bacteria, infected macrophage monolayers were washed with phosphate buffered saline (PBS) and lysed with 0.1 ml of 0.1% Triton X-100 (Sigma) to release intracellular bacteria. The intracellular *M*. *marinum* cells were fixed on glass slides and photographed utilizing an EVOS FL color imaging system (Invitrogen). Images were analyzed using ImageJ software. An MOI of 10 was applied only for the measurement of phagocytosis ratio of *M*. *marinum*.

### DAPI Staining to Detect the Nucleoid of *M*. *marinum*

Experiments were performed as referred to a published paper [23,26]. Briefly, WT and Δ*glpX* strains were cultivated to log phase in 7H9 broth supplemented with 0.2% v/v glycerol, harvested by centrifugation, washed in PBS, fixed in 4% paraformaldehyde (PFA) and stained with 0.2 μg/ml DAPI for 15 min. 5 μl stained culture was smeared on a glass slide mounted with a coverslip. Florescence signals were detected by CLSM at an excitation wavelength of 375 nm. Images were analyzed using LAS AF Lite software.

### Colony Morphology and Antimicrobial Susceptibility Assay

Experiments were performed similarly as described in a paper by *Wang et*.*al* [25]. For colony morphology observation, log phase *M*. *marinum* cultures were diluted to an OD_600_ of 0.01, and 5 μl of each dilution was incubated on 7H10 agar plates. The colonies formed after 7 days of cultivation, and colony morphology was photographed. For antimicrobial susceptibility assay, *M*. *marinum* strains were cultured in 7H9 broth with 10% OADC in 96-well plates. Selected antimicrobials were added into cultures at serial two-fold dilutions. The minimal inhibitory concentration (MIC) was defined as the minimal concentration at which no visible growth was observed after 7 days of cultivation.

## Results

### A Functional Gluconeogenic Pathway Is Dependent on *glpX*

**Fig 1:**
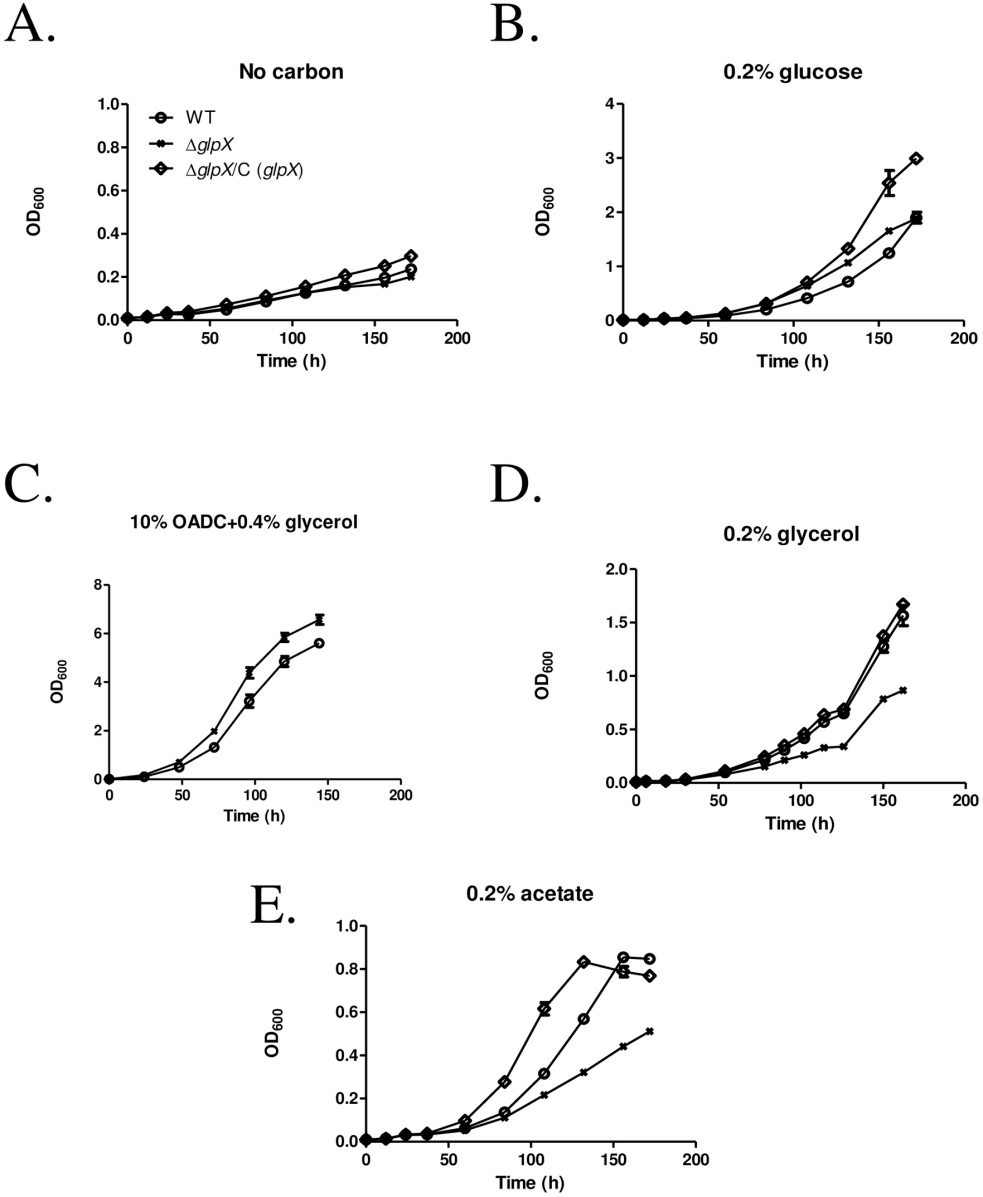
*M*.*marinum* Δ*glpX* grows on gluconeogenic and glycolytic carbon sources. WT, Δ*glpX* and complemented strain Δ*glpX*/C (*glpX*) were cultivated in 7H9 broth containing (A) no carbon source; (B) 0.2% glucose; (C) 10% OADC + 0.4% glycerol; (D) 0.2% glycerol; (E) 0.2% acetate. Data are representative of three independent experiments.

MMAR_4367, the annotated *glpX* gene in *M*. *marinum* was successfully deleted by homologous recombination as illustrated in S1 Fig. Both PCR and qRT-PCR analyses were applied to validate the mutant strain, Δ*glpX*. We first investigated the effects of *glpX* ablation on carbon metabolism of *M*. *marinum*. WT, Δ*glpX* and the complemented strain Δ*glpX*/C (*glpX*) were cultivated in 7H9 broth without or with supplementation of glycolytic (glucose) or gluconeogenic (glycerol or acetate) carbon substrates. WT and Δ*glpX* grew similarly in 7H9 broth without additional carbon source (Fig 1A**)**, or with a glycolytic carbon source, 0.2% glucose (Fig 1B), or enriched with OADC and 0.4% glycerol (Fig 1C). However, Δ*glpX* grew considerably slower than WT in 7H9 broth supplemented with 0.2% glycerol or acetate as gluconeogenic carbon sources (Fig 1D and 1E), and the complementation by expressing *M*. *marinum glpX* rescued these growth defects in Δ*glpX*.

**Fig 2:**
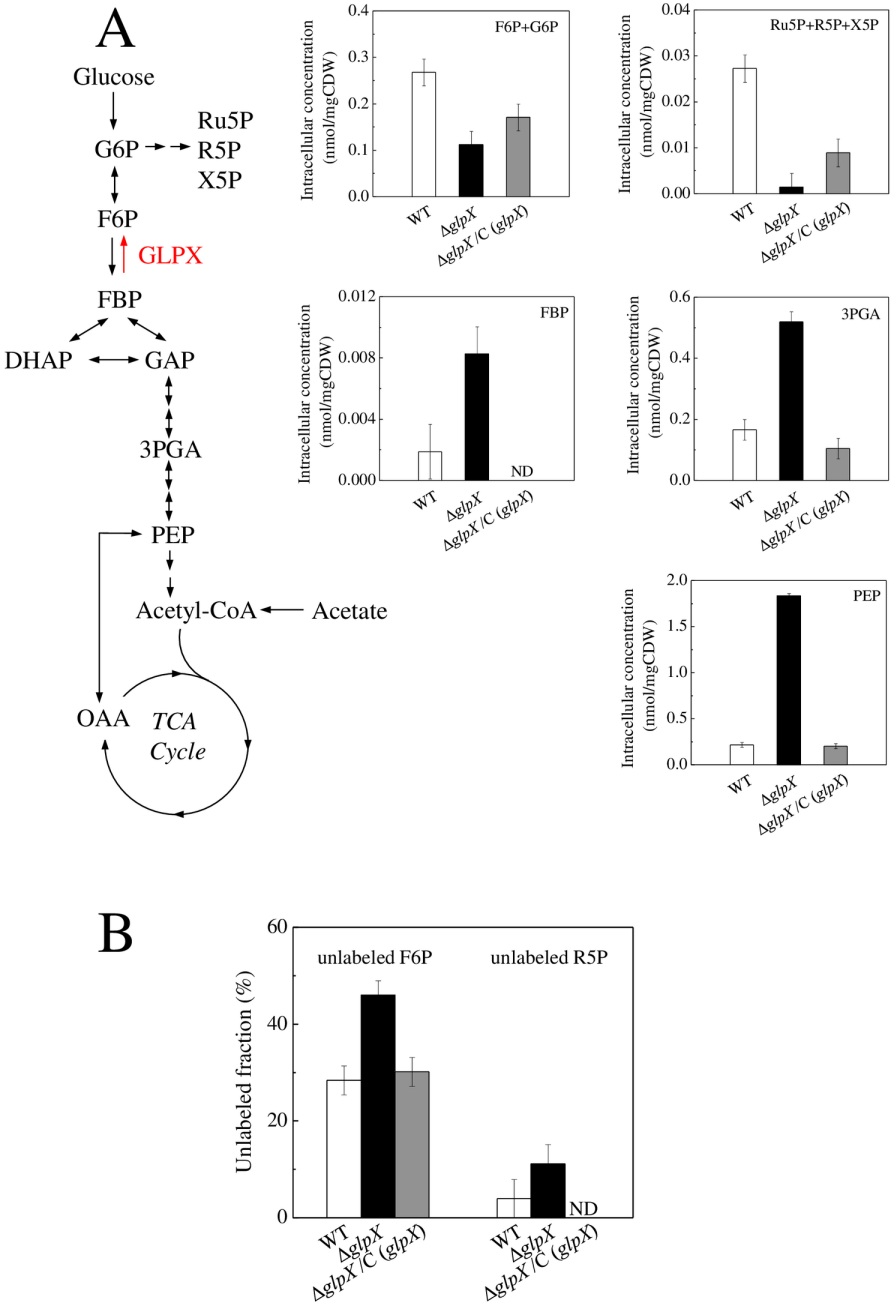
Effect of *glpX* disruption on gluconeogenic metabolism in *M*. *marinum*. The intracellular concentration (A) and unlabeled fraction (B) of metabolites in WT, Δ*glpX*, and complemented strain Δ*glpX*/C (*glpX*) were determined by LC-MS. The strains were grown on the minimal medium containing 1 g/L of [U-^13^C]acetate and harvested at an OD_600_ of 1.0. Data represent means ± standard deviation of three independent experiments. ND, not detectable. G6P, glucose 6-phosphate; F6P, fructose 6-phosphate; FBP, fructose 1,6-bisphosphate; DHAP, dihydroxyacetone phosphate; GAP, glyceraldehyde 3-phosphate; 3-PGA, 3-phosphoglycerate; PEP, phosphoenolpyruvate; PYR, pyruvate; OAA, oxaloacetate; Ru5P, ribulose 5-phosphate; R5P, ribose 5-phosphate; X5P, xylulose 5-phosphate.

To further investigate the effect of *glpX* deletion on carbon metabolism in *M*. *marinum*, we compared the intracellular concentration and ^13^C labeling of metabolites among WT, Δ*glpX* and the complemented strain Δ*glpX*/C (*glpX*). The strains were grown on the minimal medium containing 1 g/L of [U-^13^C] acetate or 1 g/L of [U-^13^C] glucose and harvested at an OD_600_ of 1.0. After rapid quenching and extraction, the metabolites upstream and downstream of the FBPase-catalyzed reaction in gluconeogenesis were analyzed by LC-MS. When grown on acetate as the sole carbon source, the intracellular concentration of the FBPase substrate fructose-1, 6-bisphosphate (FBP) in Δ*glpX* was about 4-fold higher than that in WT (Fig 2A). The 3-phosphoglycerate (3-PGA) and phosphoenolpyruvate (PEP) levels in Δ*glpX* were also increased about 3-fold and 8-fold, respectively, compared to the WT. On the other hand, Δ*glpX* showed decreased pool sizes of downstream metabolites including fructose-6-phosphate (F6P) and glucose-6-phosphate (G6P). The intracellular concentration of PPP intermediates ribulose-5-phosphate (Ru5P), ribose-5-phosphate (R5P) and xylulose-5-phosphate (X5P) in Δ*glpX* were also reduced over 10-fold compared with WT. Moreover, incorporation of ^13^C from [U-^13^C] acetate into F6P and R5P was decreased (Fig 2B). These results indicate that the gluconeogenic carbon flow is blocked at the FBPase reaction in Δ*glpX*. Complementation of Δ*glpX* by expressing *M*. *marinum glpX* led to a decrease in upstream metabolite concentrations and increases in the pool sizes and fractional ^13^C labeling of downstream metabolites (Fig 2A and 2B), indicating the restoration of the gluconeogenic flux through FBPase upon expression of *glpX*. When Δ*glpX* and WT strains were grown on glucose as the sole carbon source, no significant differences in intracellular concentrations and ^13^C labeling of the central pathway intermediates were observed (S2 Fig). This is consistent with the similar growth rate of WT and Δ*glpX* on glucose and indicates that glycolysis is not affected in Δ*glpX*. Therefore, the results of metabolite profiling and ^13^C labeling strongly suggest that *glpX* inactivation caused a disruption of gluconeogenesis at the FBPase reaction, resulting in an impairment of growth on gluconeogenic carbon sources.

### *M*. *marinum* Δ*glpX* Is Attenuated in Zebrafish

**Fig 3:**
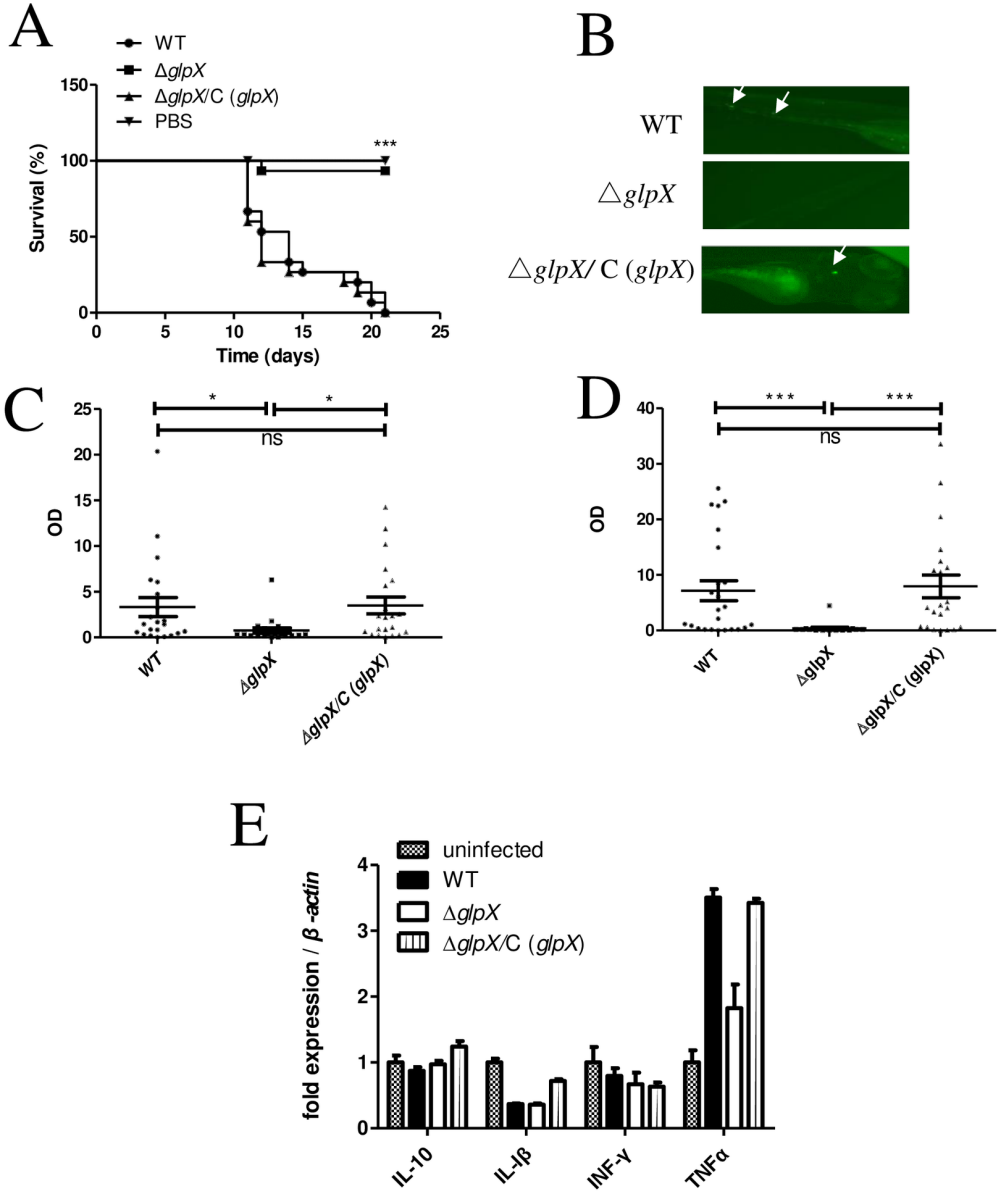
*M*.*marinum* Δ*glpX* is attenuated in zebrafish. (A) Survival curves of adult zebrafish during a 21-day infection course. Fish were intraperitoneally infected at an initial dosage of 10^4^ CFU/fish of WT, Δ*glpX*, and complemented strain Δ*glpX*/C (*glpX*). (B) Zebrafish larvae were infected with WT (56 CFU), Δ*glpX* (168 CFU) and complemented strain Δ*glpX*/C (*glpX*) (26 CFU) carrying pTEC15 containing fluorescent protein Wasabi separately. Representative fluorescent images of infected larvae at 5 dpi. The arrow points to the formation of initial granuloma with fluorescent *M*. *marinum* inside. Bacterial burdens of infected larvae were determined by FPC at 4 dpi (C) and 6 dpi (D), OD = lg^255/PixelValue^, P values are calculated by an unpaired Student *t* test. (E) The relative transcription of inflammatory cytokines were measured by qRT-PCR using total RNA extracted from zebrafish. 0.01<*P≤0.05, 0.001<**P ≤0.01, ***P ≤0.001 by Kruskal-Wallis test and Dunn's Multiple Comparison Test.

To investigate the role of *glpX* in mycobacterial pathogenesis, a zebrafish infection model was utilized [27]. Adult zebrafish were infected at an initial dosage of 1×10^4^ colony forming unit (CFU) per fish. As shown in Fig 3A, the median lethal time (LT_50_) of WT infected zebrafish was 12 days, whereas only one fish died in the Δ*glpX* group throughout the whole experimental period (21 days). The complementation by expressing *M*. *marinum glpX* rescued the virulence defects of Δ*glpX*, and the survival curve of infected zebrafish was indistinguishable from the WT infected group.

To further inspect if *glpX* subserves *M*. *marinum* to resist host innate immunity, 30 hpf zebrafish larvae were infected with WT, Δ*glpX* and the complemented strain Δ*glpX/*C (*glpX*) at initial dosages of 56, 168, and 26 CFU per fish separately. The proliferation of *M*. *marinum* was measured by FPC analysis, and expression of several pro-inflammatory or anti-inflammatory cytokines was examined by qRT-PCR, including IL-1β, IFNγ, TNFα, and IL-10. Representative pictures of infected larvae were illustrated in Fig 3B. WT proliferated normally in infected larvae, whereas Δ*glpX* displayed a significantly decreased bacterial load and defective proliferation in zebrafish larvae. Complementation by expressing *M*. *marinum glpX* restored the virulence of Δ*glpX* (Fig 3C and 3D). On 6 dpi, fish were lysed and total RNA was extracted for measuring cytokine levels. Among the tested cytokines, the elevation of TNFα transcription in zebrafish infected by WT was 3.5 fold relative to the levels seen in uninfected fish, which indicates a strong inflammatory response of the host post infection (Fig 3E). By contrast, TNFα transcription in zebrafish infected by Δ*glpX* was only 1.8 fold normalized to uninfected fish, displaying a significant drop compared to zebrafish infected by WT. Expression of in zebrafish infected by Δ*glpX/*C (*glpX*) was 3.4-fold normalized to uninfected fish, a similar level to zebrafish infected by WT. For the other three cytokines tested, no significant induction was observed. The difference of TNFα expression might reflected the different bacterial burden of zebrafish larvae infected with various strains.

### *glpX* Is Required for *M*. *marinum* Proliferation in Macrophages

**Fig 4:**
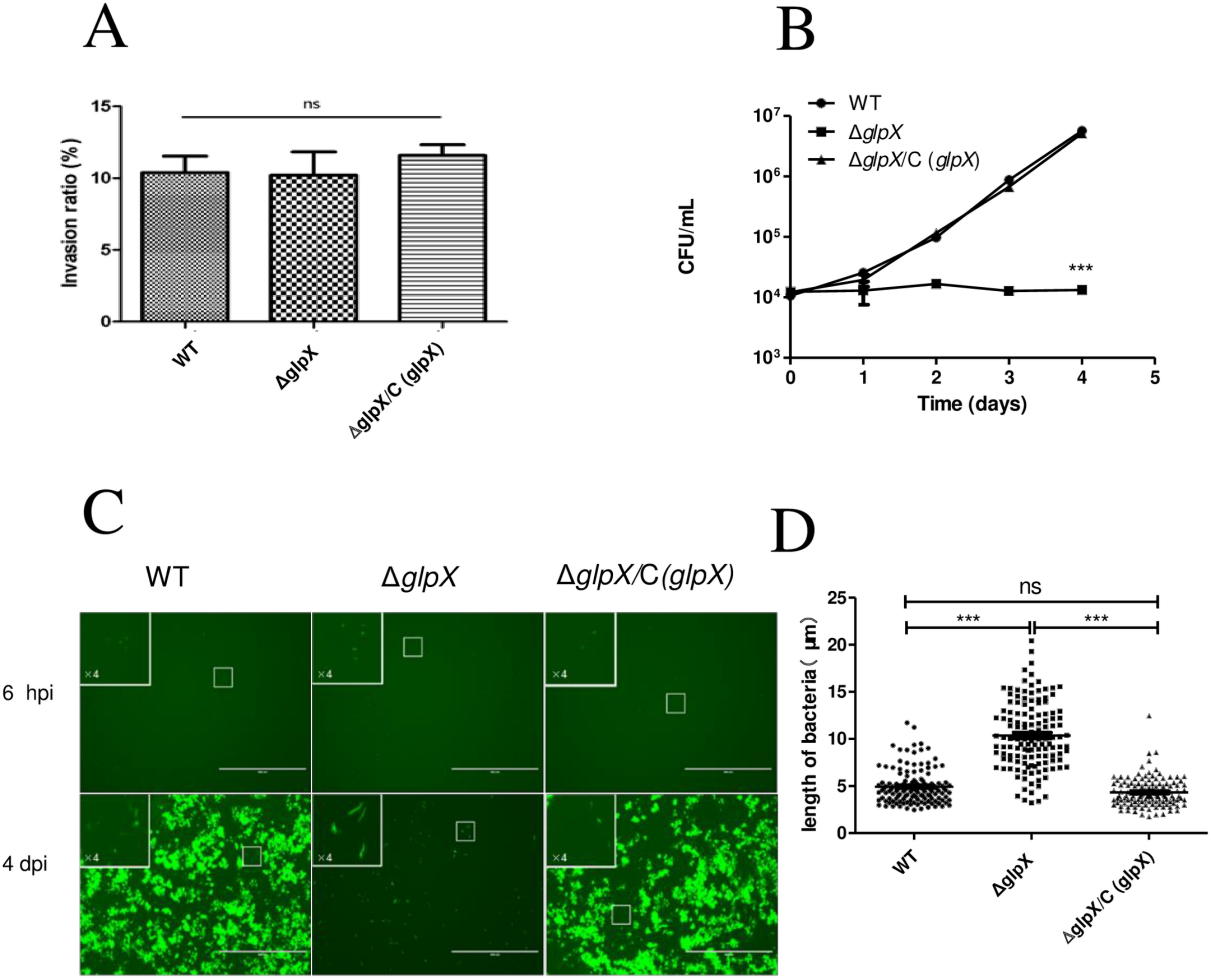
*M*. *marinum* Δ*glpX* is attenuated intracellularly and displays an elongated morphology. (A) Phagocytosis ratio of WT, Δ*glpX*, and complemented strain Δ*glpX*/C (*glpX*) into RAW264.7 macrophages, MOI = 10. (B) Replication of *M*. *marinum* strains in RAW264.7 macrophages, MOI = 1. Macrophages were lysed at the indicated time points post infection, and intracellular bacterial load was measured by CFU counts. Data represent means ± standard deviation of three independent experiments. (C) Representative images of *M*. *marinum* strains in RAW264.7 macrophages infected at MOI = 1. Scale bar, 200 μm. (D) The intracellular length measurement of *M*. *marinum*. Infected RAW264.7 macrophages (MOI = 1) lysed at 3 dpi, and length measurement is performed using the software Image J. (WT, *n* = 135 cells; Δ*glpX*, *n* = 132 cells; Δ*glpX*/C (*glpX*), *n* = 121 cells). ***P ≤0.001 by Kruskal-Wallis test and Dunn's Multiple Comparison Test.

The innate immune system is crucial for host to detect invading *Mtb*, and macrophage is a primary cell of the innate immune system and the major site of residence for *Mtb* [28,29]. To further investigate how *glpX* assists mycobacterium coping with innate immunity, macrophages were infected by *M*. *marinum* strains and two key events during infection were examined, the phagocytosis of mycobacterium and the proliferation of intracellular bacilli. It was demonstrated that the inactivation of *glpX* did not affect the phagocytosis ratio of *M*. *marinum* by RAW264.7 macrophage cells (Fig 4A), with a phagocytosis ratio of 10% similar to WT and Δ*glpX/*C (*glpX*). Intracellular proliferation of *M*. *marinum* was monitored by enumerating the bacterial load at different time points post infection. The intracellular bacterial load of WT gradually increased from 10^4^ (0 dpi) to 10^6^ CFU/ml (4 dpi), whereas Δ*glpX* lost its ability to proliferate inside macrophages yet remained at a load of 10^4^ CFU/ml (Fig 4B), the complementation by expressing *M*. *marinum glpX* rescued the proliferation defect of Δ*glpX*. Taken together, these results indicate that *M*. *marinum* proliferation inside macrophages requires a functional *glpX* gene.

### Δ*glpX* Exhibits an Elongated Morphology both *In Vitro* and *In Vivo*

It was previously reported that *Mtb* inside macrophages display an elongated morphology compared to *in vitro* cultivation, which might be correlated with intracellular stress conditions that the bacilli encounters [30]. Since altered cell length could be correlated to the change of replication rate in mycobacteria [31], we examined the morphology of Δ*glpX* inside macrophage. RAW264.7 macrophages were infected with *M*. *marinum* strains carrying a GFP containing vector pTEC15 at an MOI of 1. As shown in Fig 4C and 4D, the average length of intracellular Δ*glpX* (10.4 μm) was significantly longer than WT (4.9 μm) at 4 dpi, and the length of Δ*glpX/*C (*glpX*) was 4.3 μm.

To inspect if the elongated phenotype of Δ*glpX* is correlated with altered gluconeogenesis, *M*. *marinum* WT and Δ*glpX* were cultivated *in vitro* with the addition of glycolytic or gluconeogenic substrates. Δ*glpX* exhibited a elongated morphology when cultured in 7H9 broth containing 0.4% glycerol (S3A and S3B Fig). By contrast, no elongated morphology was observed when Δ*glpX* was cultured in 7H9 broth containing 0.4% glycerol and an additional 0.2% glucose(S3C and S3D Fig). Under both cultivation conditions, WT displayed a normal cell length. As shown in S3E Fig, the average length of Δ*glpX* (6.1 μm) was significantly longer than WT (3.1 μm) when cultured in 7H9 broth containing 0.4% glycerol. The average lengths of WT and Δ*glpX* were 2.7 and 2.4 μm respectively (S3F Fig) in 7H9 broth containing 0.4% glycerol and an additional 0.2% glucose. Collectively, these results suggest that the proliferation defects of Δ*glpX* both *in vivo* and *in vitro* is reflected by the elongated morphology and partially correlated to the altered gluconeogenesis.

### *glpX* Is Indispensable for Cell Division of *M*. *marinum* under Conditions of Gluconeogenesis

**Fig 5:**
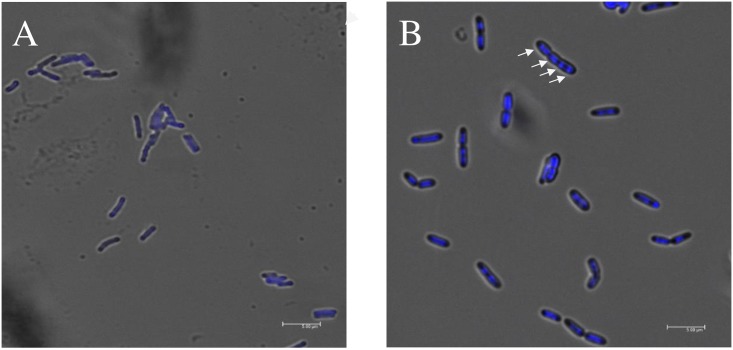
*M*. *marinum* Δ*glpX* displays altered nucleoid morphology under gluconeogenic conditions. WT (A) and Δ*glpX* (B) were cultivated in 7H9 broth containing 0.2% glycerol. Scale bar = 5 μm. Arrow indicates the typical multi-nucleoid morphology of Δ*glpX*.

In agreement with these findings, Δ*glpX* displays a multi-nucleoid (more than three) morphology when cultivated in 7H9 broth containing 0.2% glycerol (Fig 5). There are 46 out of 84 Δ*glpX* bacterial cells containing more than three nucleoids, and the percentage of this multi-nucleoid population is 54.8%. In WT, bacterial cell with over three nucleoids is not identified, except that some dividing cells contain two nucleoids. In addition, several *fts* genes which are components of either elongation complex or division apparatus were down-regulated in Δ*glpX* compared to WT and Δ*glpX/*C (*glpX*) under gluconeogenic but not enriched cultivation conditions (S4A–S4C Fig). These observations strongly suggest that *glpX* is required for the normal mycobacterial division under conditions of gluconeogenesis or during infection.

### Δ*glpX* Exhibits an Altered Antimicrobial Susceptibility

The susceptibility of *M*. *marinum* strains to various PG targeting antimicrobials were examined. MIC values of vancomycin and penicillin were 8-fold higher for Δ*glpX* compared to WT (S5A Fig), and the complementation by expressing *M*. *marinum glpX* restored the susceptibility of Δ*glpX* to WT. As a control, there was no susceptibility difference between Δ*glpX* and WT to other three tested antimicrobials, streptomycin, ethambutol, and erythromycin. In addition, Δ*glpX* exhibited a rough and dry colony morphology without the halo around the colony as in WT (S5B Fig), which also indicates possible alteration in cell wall integrity. The complementation by expressing *M*. *marinum glpX* restored the colony morphology of Δ*glpX* to WT.

## Discussion

FBPase is a crucial gluconeogenic enzyme catalyzing the irreversible conversion of FBP to F6P. Our results demonstrate that the *glpX* gene is required for the growth of *M*. *marinum* on gluconeogenic carbon sources, due to the dependency of a gluconeogenic pathway relying on FBPase encoded by *glpX*. The *glpX* gene is essential and non-redundant for the virulence of *M*. *marinum* in zebrafish. Moreover, *glpX* is needed for the normal cell morphology, cell division and proliferation of *M*. *marinum* in macrophages.

Carbon metabolism is important for intracellular bacteria to survive and proliferate in host [32], and mycobacterium displays a great flexibility in carbon metabolism [33]. F6P/G6P and PPP intermediates are vital precursors for the synthesis of cell wall components and amino acids, and the considerably reduced pool size of these metabolites in Δ*glpX* (Fig 2) might cause profound effects on mycobacterial proliferation. In support of this, the halted proliferation of Δ*glpX* inside macrophage cells (Fig 4) strengthens the importance of gluconeogenesis for *M*. *marinum* to replicate intracellularly. To understand why the intracellular proliferation ceased in Δ*glpX*, the elongated morphology (Fig 4) attracted our attention, and its correlation to gluconeogenesis was validated *in vitro* (S3 Fig). The elongated morphology is frequently correlated to dysfunction of cell division apparatus, which made us to wonder if cell division in Δ*glpX* does not function properly. This speculation was supported *in vitro* as unseparated individual cells with multi-nucleoid was only observed for Δ*glpX*, not in WT (Fig 5). Thus, the impaired gluconeogenesis led to the cessation of cell division in *M*. *marinum*. Collectively, FBPase activity is essential for normal division of *M*. *marinum* under gluconeogenic conditions and inside macrophages, and abnormal division contributes to the halted cell division upon disruption of *glpX*.

How bacteria adjust cell size and division according to its metabolic status is a fundamental yet poorly understood question. Recently, light shed on how nutritional status mediates bacterial cell size and growth rate [34]. In *Bacillus subtilis*, a conserved glucolipid biosynthesis pathway starting with the enzyme PgcA which interconverts Glc-1-P to G6P coordinates cell size with growth rate by transferring nutritional information directly to the division apparatus [35]. Additionally, defective glucosyltransferase OpgH increased the frequency of FtsZ ring formation over incompletely segregated nucleoids and reduced size of *E*. *coli* cells [36]. In both cases, UDP-glucose was proposed to indicate the nutritional status of bacteria, whose biosynthesis is dependent on G6P as the precursor during gluconeogenesis. Since the concentration of F6P/G6P was significantly lower in *ΔglpX* compared to WT growing on gluconeogenic carbon source (Fig 2), we speculated that such metabolic alteration might affect the division apparatus of *M*. *marinum*, yet to be validated. In addition, we cannot exclude the possibility that certain buildup products upon the loss of a functional FBPase may contribute to the lack of cell division in Δ*glpX*.

Two recent studies confirmed the important roles of FBPase encoding genes in gluconeogenesis and pathogenesis of *Mtb*, and concluded that *glpX* encodes an FBPase, yet the FBPase activity was not entirely eliminated in Δ*glpX* [18]. The gene *glpX* was demonstrated essential for proliferation and virulence of *Mtb* [18], and another gene *gpm2* (Rv3214) was identified to encode a novel functional FBPase [19]. Notably, MMAR_1343 which is annotated as a homologous gene to Rv3214 in *M*. *marinum*, exhibited a higher relative expression in the absence of *glpX* under gluconeogenic culture conditions (S4D and S4E Fig). It indicated that MMAR_1343 might also function as an FBPase in *M*. *marinum* upon further validation. However, the decreased growth on gluconeogenic carbon sources (Fig 1) and virulence attenuation in zebrafish (Fig 3) of Δ*glpX* indicates that MMAR_1343 cannot substitute *glpX* in *M*. *marinum*. Furthermore, the significantly attenuated virulence of Δ*glpX* in zebrafish (Fig 3) could be due to the declined bacterial proliferation *in vivo* upon the loss of *glpX*, and the lower induction of a pro-inflammatory cytokine TNFα in larvae might reflect the decreased bacterial burden. On one side, the loss of a functional FBPase turned bacilli into a non-proliferating state, which was also reported in the studies on *icl* and *pckA* mutants of *Mtb* [7,9]. On the other side, the alteration of metabolites might pose regulatory effects and finally affected the fate of *M*. *marinum*. For example, FBP may work as an effector on transcriptional regulation in model bacteria. In *E*. *coli*, FBP was proposed as a “flux-signaling metabolite” working with the transcription factor Cra during flux-dependent regulation [37]. In *B*. *subtilis*, FBP modifies DNA binding activity of a glycolytic regulator, CggR [38,39]. Whether FBP plays a direct or moonlighting role in regulatory or signaling pathways to mediate mycobacterial division *in vivo* and proliferation thereafter remains to be answered in future work. Through this, the mechanisms by which gluconeogenesis modulates mycobacterial virulence will be further elucidated.

## Supporting Information

S1 Fig Deletion and validation of *glpX* in *M*. *marinum*. (TIF)

S2 Fig Effect of *glpX* disruption on glycolytic metabolism in *M*. *marinum*. (TIF)

S3 Fig *M*. *marinum* Δ*glpX* exhibits an elongated morphology under gluconeogenic cultivation *in vitro*. (TIF)

S4 Fig *M*.*marinum* Δ*glpX* displays the altered relative expression of division genes and a second proable FBPase encoding gene, *gpm2*. (TIF)

S5 Fig *M*.*marinum* Δ*glpX* displays altered MIC and colony morphology. (TIF)

S1 Table The primers used in this study. (DOCX)
