# Supplementary figures and images for: The FBPase Encoding Gene glpX Is Required for Gluconeogenesis, Bacterial Proliferation and Division In Vivo of Mycobacterium marinum
Source: PLoS One. 2016 May 27;11(5):e0156663. doi: 10.1371/journal.pone.0156663 (PMC4883791; doi:10.1371/journal.pone.0156663)

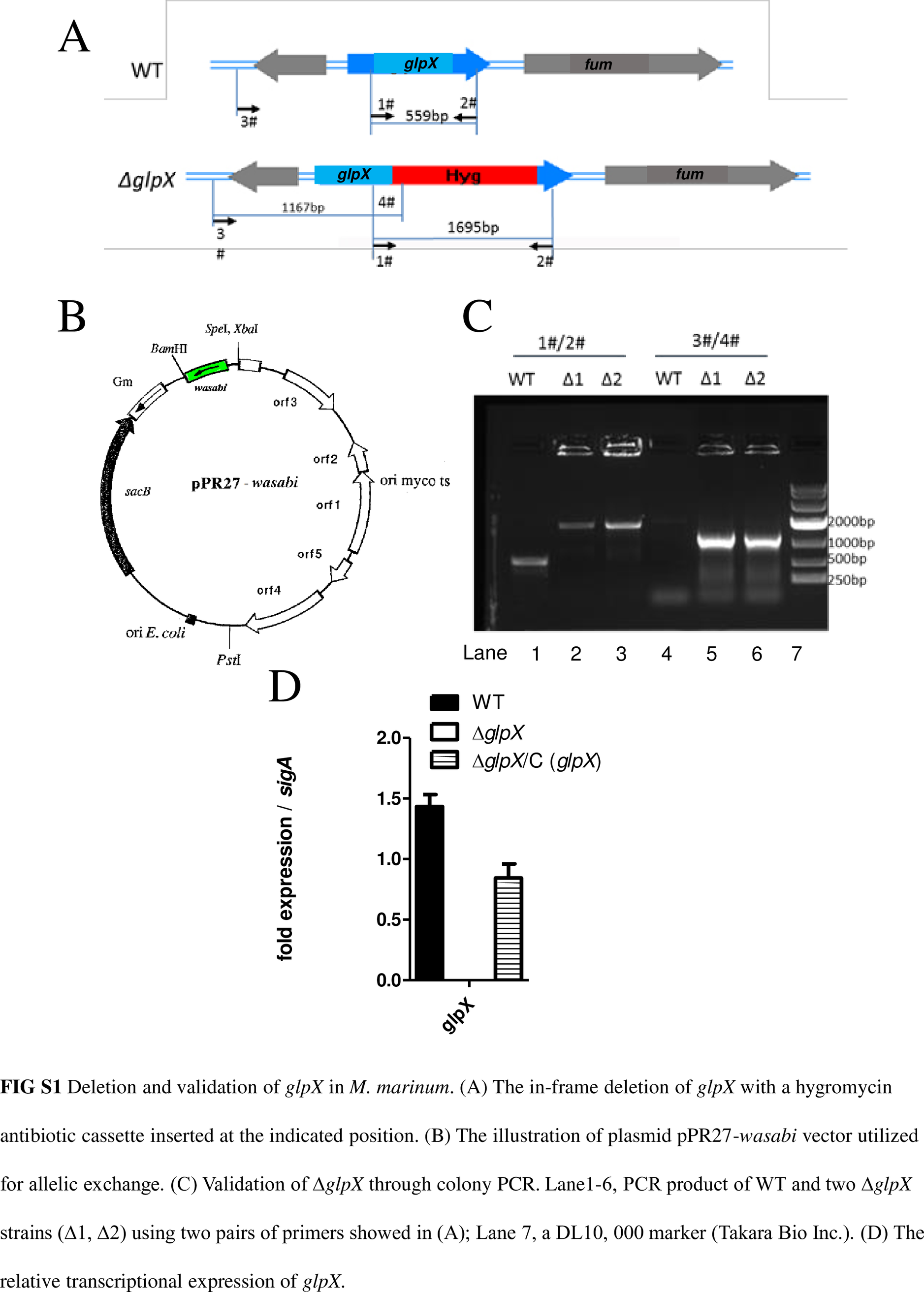

Supplement: S1 Fig — (TIF) [file pone.0156663.s001.tif]

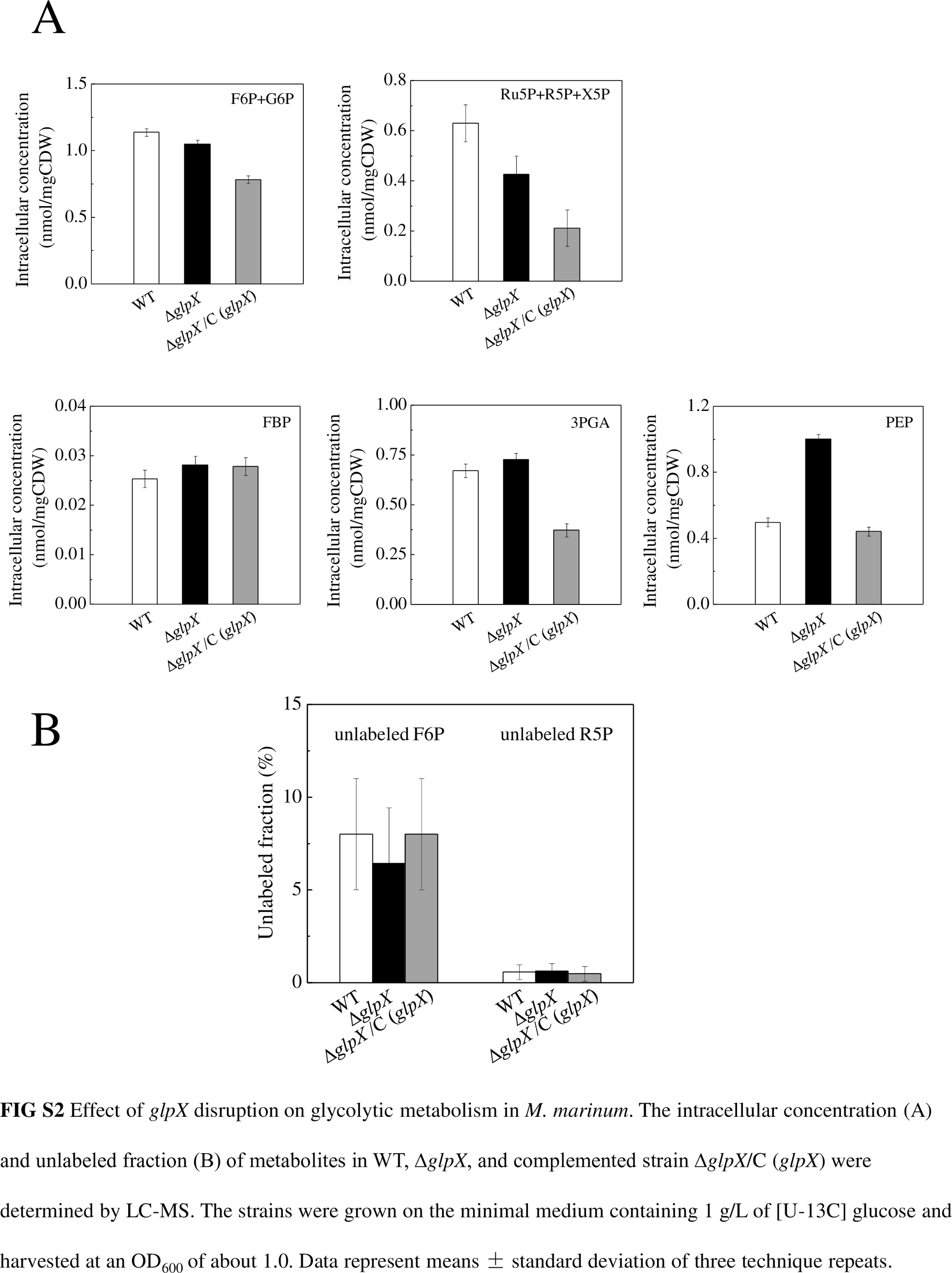

Supplement: S2 Fig — (TIF) [file pone.0156663.s002.tif]

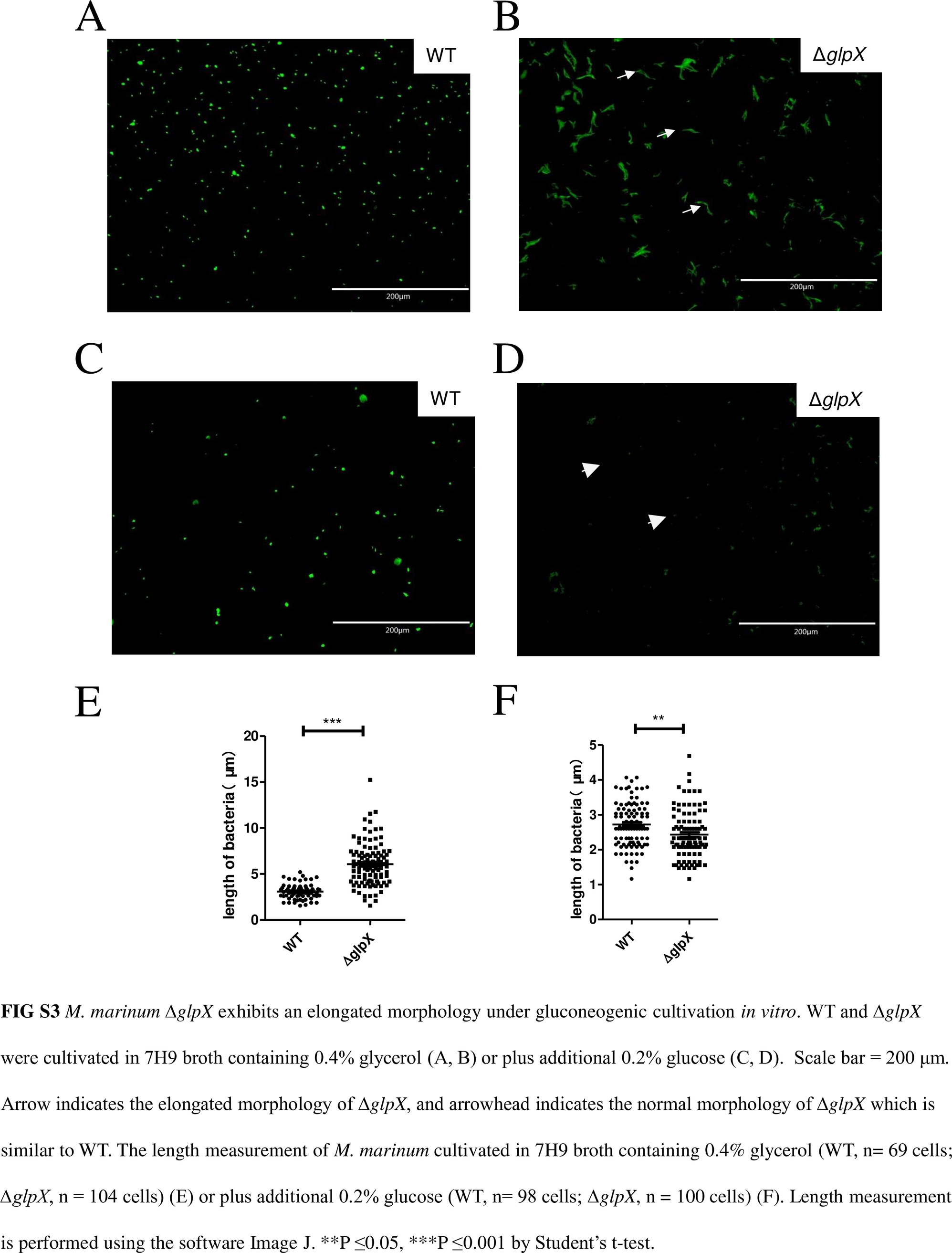

Supplement: S3 Fig — (TIF) [file pone.0156663.s003.tif]

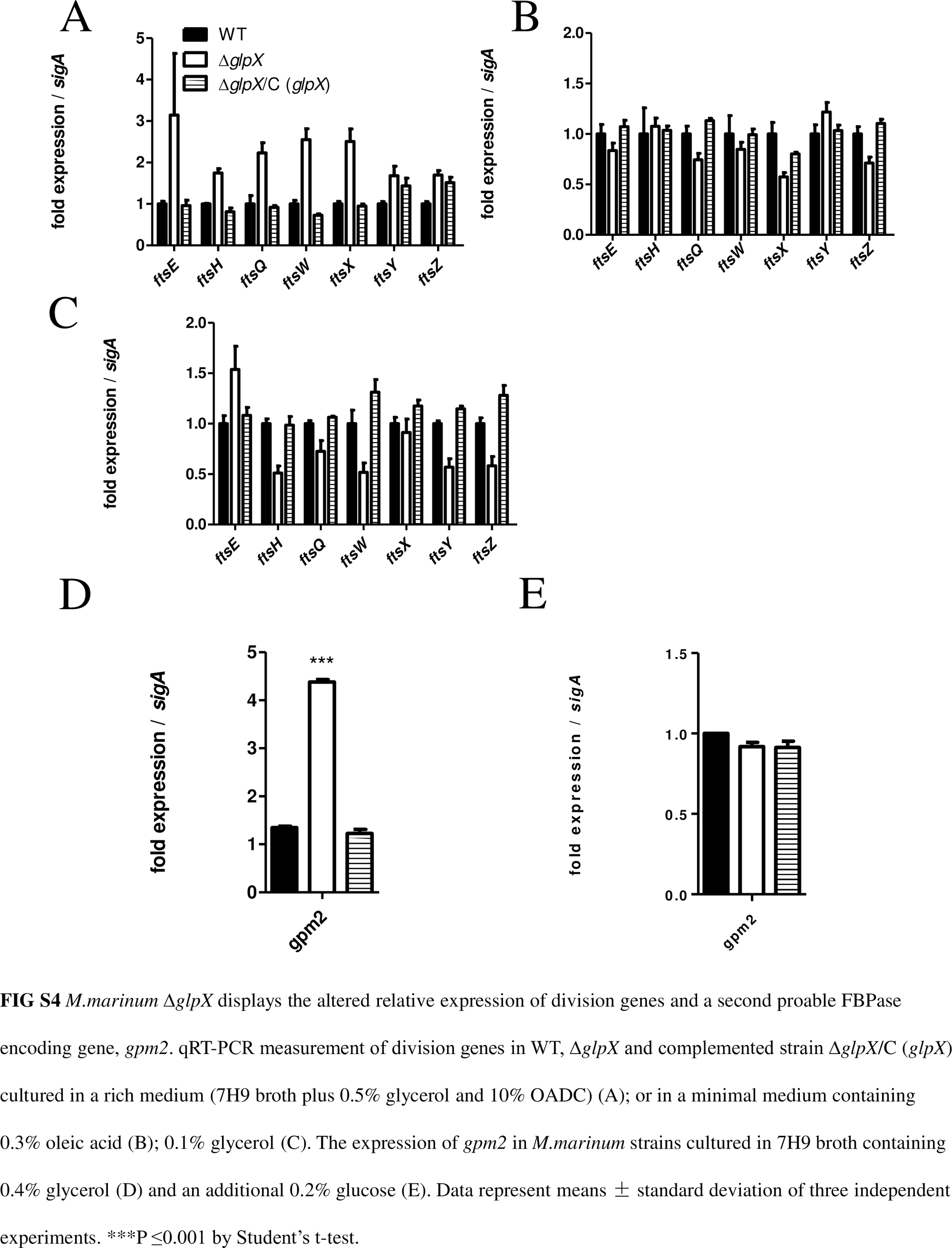

Supplement: S4 Fig — (TIF) [file pone.0156663.s004.tif]

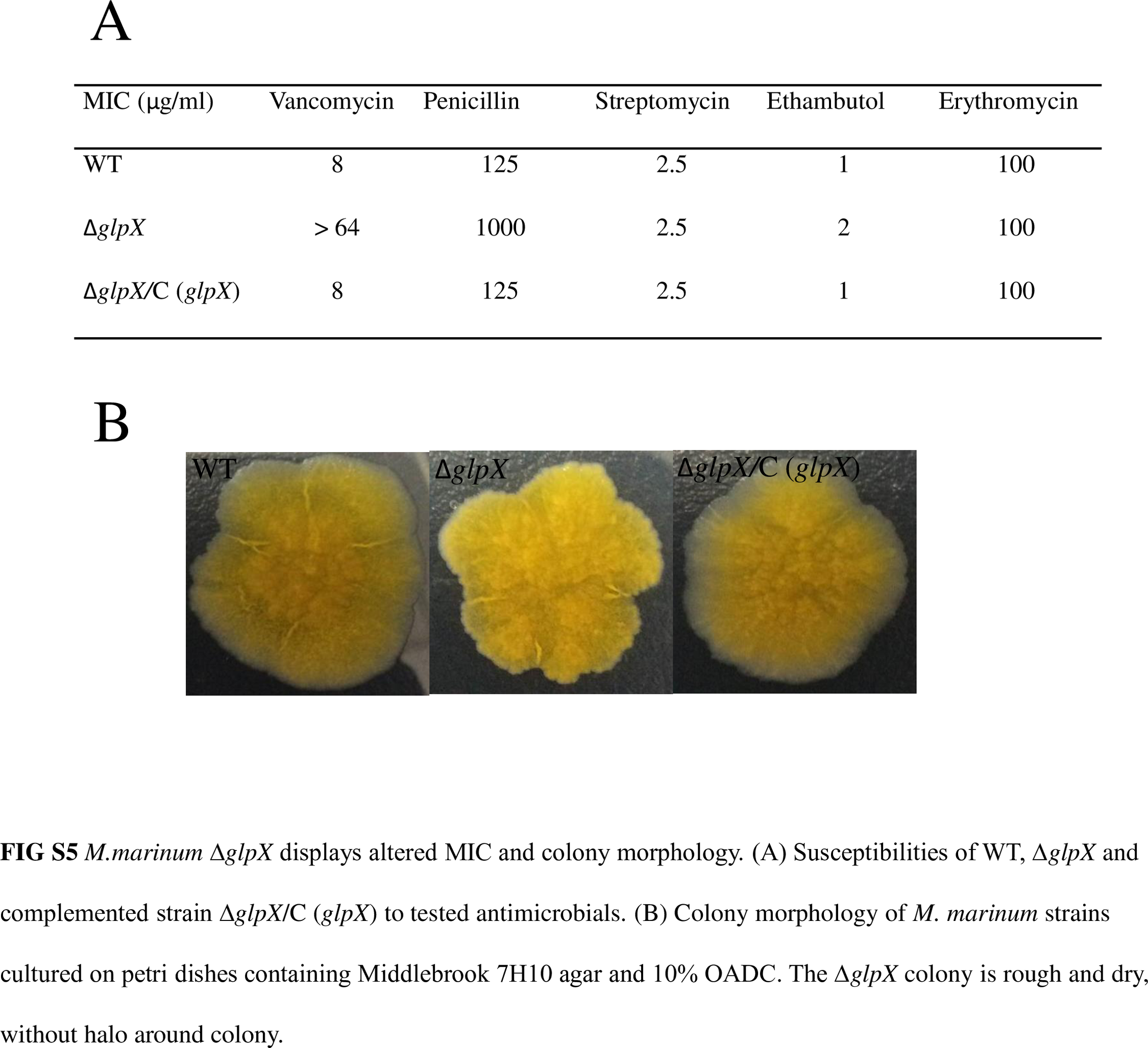

Supplement: S5 Fig — (TIF) [file pone.0156663.s005.tif]
