# Supplementary material for: The FBPase Encoding Gene glpX Is Required for Gluconeogenesis, Bacterial Proliferation and Division In Vivo of Mycobacterium marinum
Source: PLoS One. 2016 May 27;11(5):e0156663. doi: 10.1371/journal.pone.0156663 (PMC4883791; doi:10.1371/journal.pone.0156663)
Supplement: S1 Table — (DOCX) [file pone.0156663.s006.docx]

**Table S1.** The primers used in this study

| Prime name | sequence in 5′→3′direction |
| --- | --- |
| MMglpXKO_F | GGACTAGTGAGTTGGCTTTCCATCCATC |
| MM*glpX*KO_R | GCTCTAGACAATCTTGGTCAGCGAAACC |
| MM*glpX*CO_F | GGAATTCCTTGTCCTCGTCGGCATTGCTCTG |
| MM*glpX*CO_R | GACTAGTGTGTTACGGCAGGGGATAGGCG |
| GlpX_qF | GCTCAAGGGTGTTCGCTACT |
| GlpX_qR | TTGGAAAGTCGGTGGTAGGC |
| Pgm2_qF | AACTCGACAATCCGCTGGTG |
| Pgm2_qR | AGGAACCGTAATCCCACTCG |
| Pca_qF | CGCACTTCGATTCCATGCTG |
| Pca_qR | CGAATACGGAACTCGGCGAT |
| PckA_qF | ACGAGGAGTTTCATCGGCTC |
| PckA_qF | CGTCGGAGGGATCAGACAAA |
| FtsE_qF | CGACAAGGGTGAGTTCGTCT |
| FtsE_qR | GAACTTCGACACCCGCACAT |
| FtsH_qF | TCACAGATCAACGCCGACAA |
| FtsH_qR | GATGACCTTGTCGGAACCGT |
| FtsQ_qF | TGGATTTTGCGACGGGTCC |
| FtsQ_qR | CGGTCAATACCTCCAACGCA |
| FtsW_qF | GCAAAGGGGCAAACCAACTC |
| FtsW_qR | GCAGCAACGGCGATAATCAG |
| FtsX_qF | ATCTTGACGACCGCCATCTC |
| FtsX_qR | TTGGGATCGTTGGCCGATAC |
| FtsY_qF | GTCGAAGACACCCTGTTGGT |
| FtsY_qR | GTCATCCTCGCTACGGACAT |
| FtsZ_qF | TGTTGGAAGCGTCGATGGAG |
| FtsZ_qR | ATCCTGTACCAATGACGCCG |
| SigA_qF | GAAAAACCACCTGCTGGAAG |
| SigA_qR | CGCGTAGGTGGAGAACTTGT |
| TNFα_qF | AAGGAGAGTTGCCTTTACCG |
| TNFα_qR | ATTGCCCTGGGTCTTATGG |
| IL1-β_qF | TGGACTTCGCAGCACAAAATG |
| IL1-β_qR | CACTTCACGCTCTTGGATGA |
| IL10_qF | TCACGTCATGAACGAGATCC |
| IL10_qR | CCTCTTGCATTTCACCATATCC |
| IFNγ_qF | AAGATTCTCAGCTACATAATGCACACC |
| IFNγ_qR | ATGCTCATCAGTAGATTCTGCTCAC |
| β-actin_qF | ATGGATGAGGAAATCGCTGCC |
| β-actin_qR | CTCCCTGATGTCTGGGTCGTC |
